# Making meaning of multimorbidity and severe mental illness: A viewpoint

**DOI:** 10.1177/00048674231195560

**Published:** 2023-09-01

**Authors:** Sean Halstead, Dan Siskind, Nicola Warren

**Affiliations:** 1 The University of Queensland, Faculty of Medicine, Brisbane, QLD, Australia; 2 Logan Hospital, Metro South Health, Meadowbrook, QLD, Australia; 3 Metro South Addiction and Mental Health Service, Brisbane, QLD, Australia

**Keywords:** Multimorbidity, severe mental illness, comorbidity, schizophrenia, bipolar affective disorder

## Abstract

People living with severe mental illness, such as schizophrenia and bipolar affective disorder, frequently experience poorer physical health compared to those without mental illness. This issue has hitherto been approached through the disease-centred construct of *comorbidity*, where subsequent conditions are viewed as secondary to an ‘index condition’. In contrast, this *Viewpoint* sets out to explain why *multimorbidity*, a patient-centred concept that instead refers to the coexistence of multiple chronic illnesses, is a more versatile and robust framework for tackling the issue of poor physical health in people with severe mental illness. In establishing this argument, this *Viewpoint* has sought to address three key areas. First, this article will discuss the epidemiology of both physical and psychiatric multimorbidity, with respect to how they manifest at greater frequency and at younger ages in people with severe mental illness. Second, the profound consequences of this multimorbidity burden will be explored, with respect to the ‘three *D*’*s*’ of *death* (premature mortality), *disability* (functional impacts) and *deficit* (health-economic impacts). Finally, the utility of multimorbidity as a framework will be illustrated through a proposal for a three-dimensional multimorbidity construct composed of (1) quantity, (2) severity and (3) duration of an individual’s chronic illnesses. Consequently, this *Viewpoint* aims to capture why it is necessary for modern psychiatry to grasp the concept of multimorbidity to facilitate holistic healthcare for people living with severe mental illness.

## Introduction

People with severe mental illness (SMI), such as schizophrenia and bipolar affective disorder, experience significantly poorer physical health than those without SMI (Firth et al., 2019), which is associated with a greater burden of disability, frailty and premature mortality (Bendayan et al., 2022; Pearson et al., 2022; Plana-Ripoll et al., 2019a). While these consumers often have both multiple psychiatric diagnoses (*psychiatric multimorbidity*) and multiple physical health conditions (*physical multimorbidity*), the healthcare they receive is notably fragmented (Emsley et al., 2008; Langan et al., 2013). Rather than integrated treatment of both physical and mental illness, their additional health conditions outside of their SMI are frequently relegated to the status of *comorbidities*, representing separate entities that are of less significance in the hierarchy of conditions requiring treatment (Langan et al., 2013; Launders et al., 2022; Nicholson et al., 2019).

Psychiatrists are placed in a crucial position to advocate for the holistic needs of their consumers. Applying a multimorbidity framework to patient care offers a means to do this, as ‘a shift from a single disease–centric approach to a more patient-centred view’ (Launders et al., 2022: 3), where no single diagnosis has an absolute priority over others (Langan et al., 2013; Nicholson et al., 2019). This enables clinicians to conceptualise medical histories that capture multiple simultaneous conditions.

**Figure 1.**
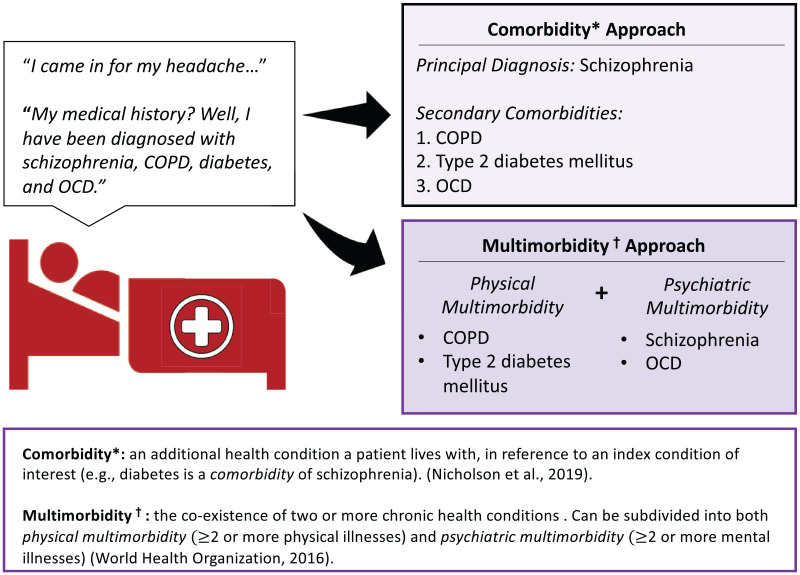
Conceptualising comorbidity against multimorbidity.

To illustrate the conceptual difference between comorbidity and multimorbidity, suppose there exists a 60-year-old hypothetical consumer, ‘Red’, who presents to a health service with a headache (Figure 1). Red has a history of schizophrenia, diabetes, chronic obstructive pulmonary disorder (COPD) and obsessive–compulsive disorder (OCD). If Red’s past medical history is viewed through a comorbidity lens, clinicians may have a tendency to prioritise one condition as the principal diagnosis and list the other conditions as secondary comorbidities. A multimorbidity framework instead places both psychiatric and physical conditions on equal footing. Here, Red can be classified as having both physical and psychiatric multimorbidity due to having two conditions in each domain. This multimorbidity approach favours a more balanced understanding of how an individual’s multiple conditions interact, enabling clinicians to better appraise the holistic health needs of the consumer.

However, as multimorbidity is still an emerging public health concept, there remains discourse over how it should be defined and measured (Johnston et al., 2018; Pearson-Stuttard et al., 2019; Xu et al., 2017). Furthermore, there is limited research on how multimorbidity should be applied to consumers with SMI.

This *Viewpoint* aims to contextualise the importance of multimorbidity to psychiatry through three questions. First, how does multimorbidity manifest in people living with SMI? Second, what are the impacts of multimorbidity in people with SMI? Third, why may the framework of multimorbidity have greater utility and benefit for those with SMI over other frameworks like comorbidity?

## Multimorbidity in people with SMI

Psychiatrists must first be aware of how multimorbidity manifests in consumers with SMI, with respect to both psychiatric and physical domains.

While it is infrequently labelled as *psychiatric multimorbidity*, the presence of multiple psychiatric diagnoses is ubiquitous in cohorts with SMI (Tsai and Rosenheck, 2013). A Danish population study by Plana-Ripoll et al. (2019b) demonstrated that all categories of mental illness exhibit bi-directional hazard ratios between one another. In particular, co-occurrence of substance use disorders with SMI is widely prevalent, and the term ‘dual diagnosis’ is frequently employed to describe consumers with this type of psychiatric multimorbidity (Hjorthøj et al., 2015; Schmidt et al., 2011). Individuals with single psychiatric diagnoses are often the exception rather than the rule (Van Loo et al., 2013).

By comparison, there is a greater paucity of research on physical multimorbidity in cohorts with SMI, even though this cohort’s increased risk of specific physical comorbidities is well elucidated (Momen et al., 2020). To date, only one published meta-analysis by Rodrigues et al. (2021) has examined the epidemiology of physical multimorbidity in cohorts with and without a psychotic disorder. This study demonstrated that people with psychotic disorders have a risk ratio of 1.69 (95% confidence interval [CI]: 1.37–2.08) of having two or more chronic physical conditions, compared to those without psychosis. While the accuracy of these results was constrained by significant heterogeneity due to varying multimorbidity definitions, higher odds of physical multimorbidity in people with SMI (odds ratio [OR]: 1.84, 95% CI: 1.80–1.88) was also demonstrated in a recent study with over 200,000 participants (Launders et al., 2022). Notably, these odds ratios were more pronounced for younger male and female cohorts (under the age of 50) and cluster analysis of multimorbidity showed a largely similar profile of disease clusters between cohorts with and without SMI. The authors concluded that while mostly the same physical conditions cause poor physical health between cohorts, physical multimorbidity manifests at greater frequency and at younger ages in those with SMI (Launders et al., 2022).

**Figure 2.**
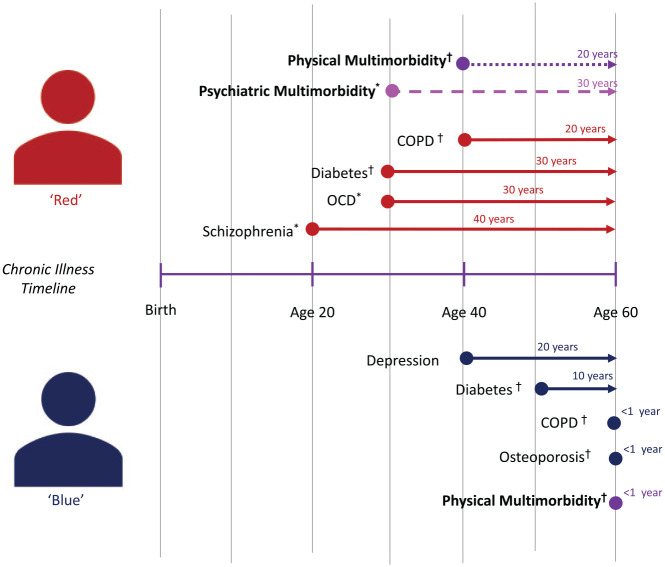
Multimorbidity progression between a person with (red) and without (blue) severe mental illness.

This increased frequency and earlier onset of multimorbidity in people living with SMI correlates to a greater lifetime exposure to poor health. There are a multitude of both non-modifiable (e.g. genetic, developmental and sociocultural factors) and modifiable (e.g. lifestyle behaviours, effects of psychotropic medication and health system factors) that contribute to this (Brasso et al., 2021; Firth et al., 2019; Zamanpoor, 2020). Understanding how the extent of multimorbidity exposure differs between those with and without SMI is central to understanding the health inequities faced by this cohort. Returning to the example of Red, suppose there is another 60-year-old consumer, ‘Blue’, who has a mild depressive illness, COPD, diabetes and osteoporosis, but no SMI (Figure 2). At first glance, both Red and Blue have four chronic conditions at the age of 60, which may be interpreted that their health needs are roughly similar under a comorbidity model. However, if their multimorbidity is instead mapped against the timeline of their diagnoses, it becomes apparent that Red has had psychiatric and physical multimorbidity for 30 and 20 years, respectively, while Blue only developed physical multimorbidity at the age of 60. Compared to a comorbidity model, this type of multimorbidity assessment suggests that consumers like Red have faced a significantly greater cumulative exposure to chronic disease, both physical and psychiatric.

## The impacts of multimorbidity in people with SMI

**Figure 3.**
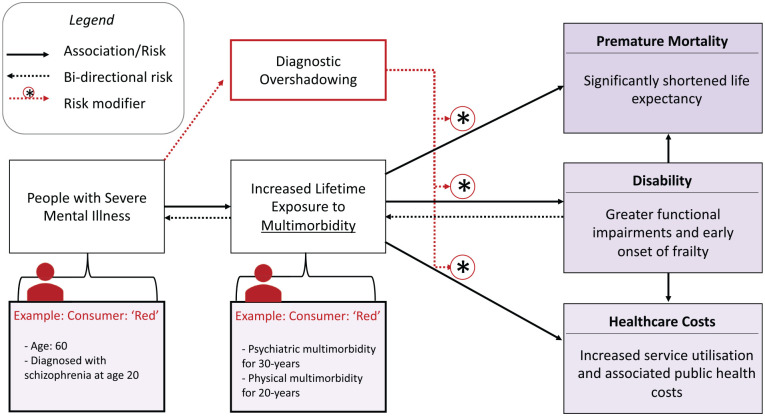
Impacts of multimorbidity on death, disability and health-economic deficit.

Recognition of multimorbidity in consumers with SMI allows psychiatrists to highlight their long-term health risks. The three principal impacts that multimorbidity has on this cohort are best summarised through the ‘three *Ds*’ of *death* (premature mortality), *disability* (functional impacts from multimorbidity) and *deficit* (health-economic consequences) (Figure 3).

### Death: premature mortality arising from multimorbidity

People with SMI have a reduced life expectancy broadly between 10 and 20 years, generally worse in males compared to females (Chesney et al., 2014; Hjorthøj et al., 2017; Laursen et al., 2016, 2019). A majority of this premature mortality is attributable to death from physical illness rather than external causes such as suicide (Plana-Ripoll et al., 2019a; Walker et al., 2015). Those with both SMI and physical disease have a significantly increased risk of mortality compared to those with physical disease alone (Davies et al., 2021). It is hypothesised that multiple physical conditions (i.e. physical multimorbidity) further compounds this mortality risk (Stubbs et al., 2016).

Diagnostic overshadowing, and the associated underdiagnosis and undertreatment of physical health, is suggested to be a substantial influence on this premature mortality (Launders et al., 2022). Misattribution of somatic symptoms to mental illness occurs for a multitude of reasons, such as stigmatising views of clinicians, difficulties in communication, challenging patient behaviour and complexity of presentations (Shefer et al., 2014). Large register studies from Denmark and Norway have demonstrated that people who lived with SMI and subsequently died from chronic physical illness were significantly more likely to have never been diagnosed with that physical illness while alive (Brink et al., 2019; Heiberg et al., 2019). Thus, while physical multimorbidity has more of a direct relationship on premature mortality, psychiatric multimorbidity compounds this risk through diagnostic overshadowing.

### Disability: functional impacts of multimorbidity

Schizophrenia and bipolar disorder are two of the leading causes of disability worldwide and it is hypothesised that this is strongly associated with early accumulation of physical illness (Strassnig et al., 2014; Weye et al., 2021; World Health Organization, 2011). Individuals with both SMI and physical multimorbidity are greater impacted on activities of daily living compared to those without physical multimorbidity (Mirza et al., 2021), potentially due to marked deficits in both physiological capabilities (e.g. work capacity, strength and cardiac output) as well as cognitive deficits (e.g. issues with memory and decision-making). This level of physiological and psychosocial dysfunction is more typically seen with ageing and development of frailty, which has been shown to be more prevalent and occur earlier in those with SMI than the general population (Pearson et al., 2022). It is important to recognise that these associations between multimorbidity and outcomes of disability are interconnected and exhibit bi-directional effects (Pengpid et al., 2022). Disability and frailty place these individuals at greater vulnerability of worsening physical and mental health in a positive feedback loop. This vicious cycle is exacerbated by diagnostic overshadowing, as increased complexity makes it difficult for these individuals to receive the holistic care needed for their multimorbidity.

### Deficit: the healthcare costs of multimorbidity

Multimorbidity acts as a compounding influence on healthcare cost, whereby each additional chronic condition is associated with an almost exponential rise in cost (Lehnert et al., 2011). As a cohort with a high healthcare cost, it is suspected that multimorbidity is a significant mediator of why this is the case for consumers with SMI (Zhang et al., 2020). Studies from the Netherlands and India have both demonstrated that the presence of simultaneous physical and mental illness is responsible for greater healthcare utilisation and costs, compared to people with mental illness alone (Pati et al., 2020; Van Schijndel et al., 2022).

Multiple factors likely contribute to this phenomenon. First, those with simultaneous physical and mental illness undergo more frequent re-admission to hospital (Šprah et al., 2017), and are more likely to have adverse discharge dispositions (Benraad et al., 2020). Diagnostic overshadowing and restrictions in accessibility can result in undertreated physical illness requiring avoidable emergency department visits and longer admissions (Van Schijndel et al., 2022). Moreover, consumers with multimorbidity can face larger amounts of diagnostic testing and more prescriptions (Pati et al., 2020), resulting in polypharmacy (Lunghi et al., 2020). Finally, the primary care sector faces a diverse set of challenges when treating consumers with SMI. Some consumers have increased primary care utilisation which has resource allocation impacts (Brink et al., 2019), while others face reduced primary care access due to difficulties in managing appointments, concerns of stigma and symptoms of avolition and paranoia (Waterreus and Morgan, 2018).

## Clinical utility of the multimorbidity framework to psychiatry

Psychiatrists have a crucial role to play in utilising the framework of multimorbidity to improve the health outcomes of consumers with SMI. Compared to a comorbidity model, a multimorbidity framework enables psychiatrists to better capture the simultaneous physical and mental health needs of their consumers. Subsequently, this framework empowers psychiatrists to more effectively advocate the need for integrated care models.

### Evaluating multimorbidity: a framework proposal for measuring ‘illness-years’

Multimorbidity has traditionally been evaluated through disease counts, with examples such as the Charlson Comorbidity Index (CCI) (Charlson et al., 1987) and the Elixhauser count (Elixhauser et al., 1998). However, these indices only tally the presence of several specific diseases at a singular point in time and do not capture longitudinal exposure to disease, nor have they been designed for psychiatric cohorts.

**Figure 4.**
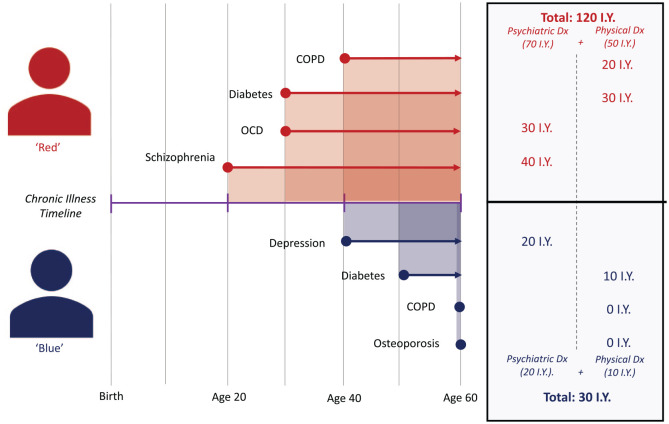
Application of ‘illness-years’ (IY) to evaluate chronic illness exposure.

Instead, we propose that multimorbidity is evaluated through ‘illness-years’ (IY), a novel three-dimensional variable based upon (1) quantity of chronic diseases, (2) the severity of chronic disease and (3) the accumulated duration of each chronic condition. This is analogous to how smoking exposure is quantified in pack-years as a composite of duration and quantity (Bernaards et al., 2001). In Figure 4, we have applied this to the consumers Red and Blue and calculated their total IY’s exposure by taking the sum of the individual durations of each of their chronic illnesses across their lifetime. As depicted, while both individuals have the same number of chronic illnesses at the age of 60, Red’s total IY of chronic disease (120 IY) is four times greater than Blue’s (30 IY). We hypothesise that depicting multimorbidity in this fashion could enable greater clinical prognostication of key health outcomes, similar to how pack-years of smoking has been used to prognosticate risk of COPD and lung cancer (Çolak et al., 2021; Janjigian et al., 2010).

Greater precision could be introduced by weighting each chronic condition by its severity, so that, uncontrolled chronic conditions are potentially weighted more heavily for each year they persist. To our knowledge, this type of composite measure has not been used previously in studying multimorbidity in people with SMI. Maciejewski and Hammill (2019) used a similar concept to IYs by measuring ‘cumulative duration’ (sum of years of exposure for 19 different chronic conditions) to examine the association between multimorbidity and health expenditure in a geriatric cohort; similar to the CCI, only a select list of conditions were measured, whereas IY aims to be less restrictive.

Quantifying multimorbidity in a manner sensitive to number and duration of chronic illness enables for a more versatile framework to capture the holistic health of consumers with SMI. Application of the concept could allow psychiatrists to stratify the chronic disease exposure of their consumers (as shown in Figure 4) and better determine the level and types of health interventions required.

### Managing multimorbidity: advocating for integrated care

In translation of multimorbidity to clinical practice, psychiatrists have a fundamental duty to advocate for integrated care of physical and mental health.

**Figure 5.**
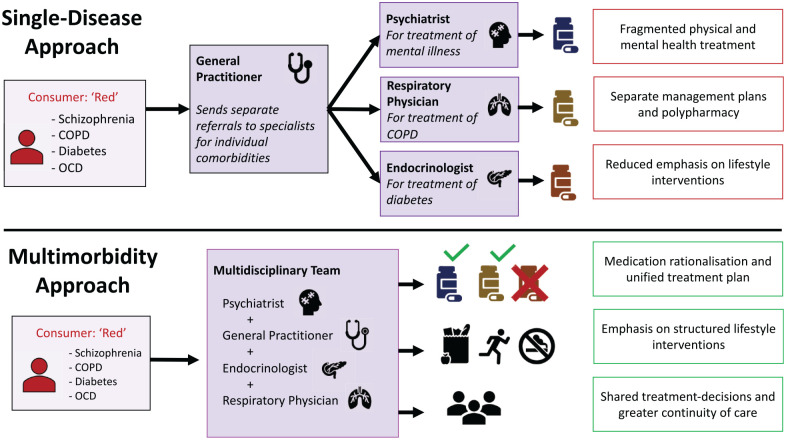
Comparison of single-disease and multimorbidity care models. “Graphic Sources: Pancreas graphic in figure 5 was a royalty-free image by illustrator F. Akbar obtained with a free Digital License from https://iconscout.com. All other graphics were royalty-free stock icons available for use from the Microsoft 365 License.”

Most existing models of care are typically based on a ‘single disease approach’ whereby a general practitioner (GP) makes separate referrals to various specialists for management of individual comorbidities (Langan et al., 2013). For a consumer like Red, the involvement of multiple different specialists can lead to fragmented care whereby their mental health is treated by their psychiatrist, their COPD by their respiratory physician and their diabetes by their endocrinologist (‘Single-disease approach’ in Figure 5). This single-disease approach treats each condition in isolation, which fails to appreciate the diverse and interwoven needs of consumers. Instead, we recommend a multidisciplinary approach to care of multimorbidity. Under this model, if Red’s health practitioners instead convened and had shared partnership over major treatment decisions, this would facilitate a unified management plan that aimed to improve both Red’s physical and mental health (‘Multimorbidity approach’ in Figure 5). This type of care model is not a new proposal, as there are some long-established multidisciplinary clinics which emulate this concept, such as the Sydney-based ‘Collaborative Centre for Cardiometabolic Health in Psychosis’ (Kritharides et al., 2017). Such examples can illuminate how multimorbidity can be embraced in clinical practice.

In the prevention and management of both physical and psychiatric multimorbidity, integrated physical and mental healthcare interventions are an imperative, such as structured lifestyle interventions that target major risk factors for both domains, like diet and physical activity (Firth et al., 2019). Judicious use of pharmacological interventions will also help apply brakes to unnecessary polypharmacy in consumers with multimorbidity. Medication rationalisation tools, such as OPTIMISE by Carolan et al. (2022), can help achieve this through a systematic, evidence-based assessment of the need for pharmacological treatment.

The size and direction that multidisciplinary multimorbidity care teams take will be dependent upon the extent of multimorbidity faced by each individual consumer. For young consumers with newly diagnosed SMI and no other illnesses (i.e. few IY), the emphasis will be placed on primary prevention and screening of multimorbidity, which could be delivered through shared partnership between a psychiatrist and GP. For consumers like Red with extensive IY, tertiary prevention of complications (such as the three Ds) will instead be critical, requiring the additional involvement of multiple subspecialists and allied health professionals. Leadership and coordination of multidisciplinary care is fundamental, and for many consumers with SMI, psychiatrists are optimally positioned to act as navigators and gatekeepers for multimorbidity care, particularly for those with complex and treatment-refractory SMI requiring more frequent service engagement. However, this role may shift between practitioners (e.g. psychiatrists, general practitioners and allied health professionals) depending upon the health needs, preference of the consumer and availability of services.

## Conclusion

Multimorbidity is an emerging public health concept of huge relevance to psychiatry. Through summarising the pertinent literature, this *Viewpoint* has sought to address the following key points. First, because people with SMI are exposed to a greater burden of biological, psychological, social and lifestyle risk, they are placed at greater risk of accumulating multiple chronic conditions (i.e. multimorbidity). Second, the presence of multiple physical conditions (physical multimorbidity) in conjunction with multiple psychiatric diagnoses (psychiatric multimorbidity) leads to diagnostic overshadowing, whereby increased patient complexity can result in the underdiagnosis and undertreatment of physical disease. Third, increased rates of untreated multimorbidity result in multiple profound impacts, such as premature mortality, increased disability and increased healthcare needs. Importantly, these phenomena function not only in a unidirectional current, but instead form a positive feedback loop, where end outcomes such as disability and frailty constitute additional risk factors for developing further health conditions, leading to increased severity of multimorbidity.

Multimorbidity is a potent, holistic and versatile framework, and future research should strive to implement it to better understand its manifestation and impacts in people with SMI. Moreover, use of dynamic and multidimensional measurements of multimorbidity that consider duration, quantity and severity of chronic disease are likely to be more comprehensive, capturing a more accurate representation of an individual’s risk to poor health outcomes. Now moving away from a ‘single-disease’ approach to medicine, psychiatrists have a lead role to play in applying the concept of multimorbidity to their clinical practice to advocate for integrated physical and mental healthcare.
